# Th22 cells and the intestinal mucosal barrier

**DOI:** 10.3389/fimmu.2023.1221068

**Published:** 2023-08-14

**Authors:** Jieli Chen, Jun Yao

**Affiliations:** ^1^ Department of Gastroenterology, The Second Clinical Medical College, Jinan University, Shenzhen, Guangdong, China; ^2^ Department of Gastroenterology, Shenzhen People’s Hospital, Shenzhen, Guangdong, China

**Keywords:** Th22, IL-22, intestinal mucosal barrier, intestinal homeostasis, inflammatory bowel disease

## Abstract

T-helper 22 (Th22) cells represent a novel subset of CD4^+^ T cells that exhibit distinctive characteristics, namely the secretion of IL-22 while abstaining from secreting IL-17 and interferon-γ (IFN-γ). These cells serve as the primary source of IL-22, and both Th22 cells and IL-22 are believed to play a role in maintaining intestinal mucosal homeostasis in inflammatory bowel disease (IBD). However, the precise functions of Th22 cells and IL-22 in this context remain a subject of debate. In this work, we aimed to elucidate their impact on the integrity of the intestinal mucosal barrier by presenting an overview of the molecular structure characteristics and functional effects of Th22 cells and IL-22. Furthermore, we would explore targeted treatment approaches and potential therapeutic strategies focusing on the Th22 and IL-22 pathways.

## Introduction

Inflammatory bowel disease (IBD) encompasses a collection of disorders marked by persistent inflammation within the gastrointestinal tract, notably ulcerative colitis (UC) and Crohn’s disease (CD). The global incidence of IBD continues to rise annually, with a current estimate of approximately 5 million affected individuals worldwide (1, 2). Although the precise etiology of IBD remains elusive, numerous investigations have identified three principal causative factors: individual genetic susceptibility, heightened immune activation, and compromised intestinal mucosal barrier function (3).

The intestinal mucosal barrier, encompassing the physical, chemical, immune, and biological components, holds immense significance in maintaining intestinal homeostasis and overall bodily health. It serves not only to prevent the invasion of intestinal pathogens but also to actively modulate immunity and the intestinal microbiota in a reciprocal manner (4).

In the context of IBD patients, immune cells assume a pivotal role in preserving the functionality of the intestinal mucosa. The activation of the immune response in these individuals leads to heightened intestinal permeability, disruption of the structural integrity of the intestinal barrier, and imbalance in the composition of the intestinal flora. Consequently, this triggers a more pronounced immune response. Increasingly, research is acknowledging damage to the intestinal mucosa as a fundamental mechanism underlying the onset of IBD. An aberrant immune response specifically targets the intestine, eventually precipitating changes in the intestinal milieu. Thus, it is crucial to gain a comprehensive understanding of the regulation of immune responses in relation to the intestinal barrier in order to unravel the pathogenesis of IBD.

Th22 cells, a subtype of CD4^+^T cells, have recently gained considerable attention in the field of immunology. These cells have been observed at sites of infection and in various autoimmune diseases. However, their precise molecular characteristics and functional roles remain largely unknown. Upon specific stimulation, Th22 cells secrete interleukin 22 (IL-22) while abstaining from producing IL-17 and interferon-γ (IFN-γ). Among all cell types, Th22 cells are considered the primary source of IL-22. It is widely believed that Th22 cells are associated with the intestinal mucosal barrier (5), but the specific mechanisms underlying this relationship have not been extensively explored. Consequently, our research aimed to investigate the connection between Th22 cells and the intestinal mucosal barrier, shedding light on the underlying mechanisms involved.

## Physiology of Th22 cells

Upon the initial discovery of Th22 cells, researchers have noted several similarities between these cells and Th17 cells. However, subsequent experiments have provided new insights. It is observed that a particular subset of CCR6^+^ CD4^+^ T cells, which is typically associated with Th17 cells, selectively secretes IL-22 while abstaining from producing IFNγ and IL-17. This distinctive characteristic sets Th22 cells apart from Th17 cells and justifies their classification as Th22 cells (6). Furthermore, experimental evidence demonstrates that during their development, Th22 cells do not express IL-17A-related proteins. As a result, they are recognized as a separate lineage distinct from Th17 cells (7).

Th22 cells are generated from naive CD4^+^ T cells in response to various cytokines, including IL-6, IL-23, IL-1β, and TNF-α (6, 7). Once matured, Th22 cells exhibit robust secretion of IL-22, along with concurrent production of TNF-α and IL-13. Notably, Th22 cells do not produce IL-17 or IFN-γ (6). Basu et al. have demonstrated that IL-6 is crucial for the differentiation of IL-22-producing CD4^+^ T cells (8). Although IL-23 also plays a pivotal role in promoting Th22 cell maturation, its function is mediated through IL-6 production (9). The combined action of IL-6 and IL-23 significantly enhances IL-22 secretion by Th22 cells, whereas IL-23 alone is insufficient to induce Th22 cell differentiation. Transforming growth factor-beta (TGF-β), which is indispensable for Th17 cell differentiation, exerts an inhibitory effect on Th22 cell differentiation (9, 10).

**Figure 1:**
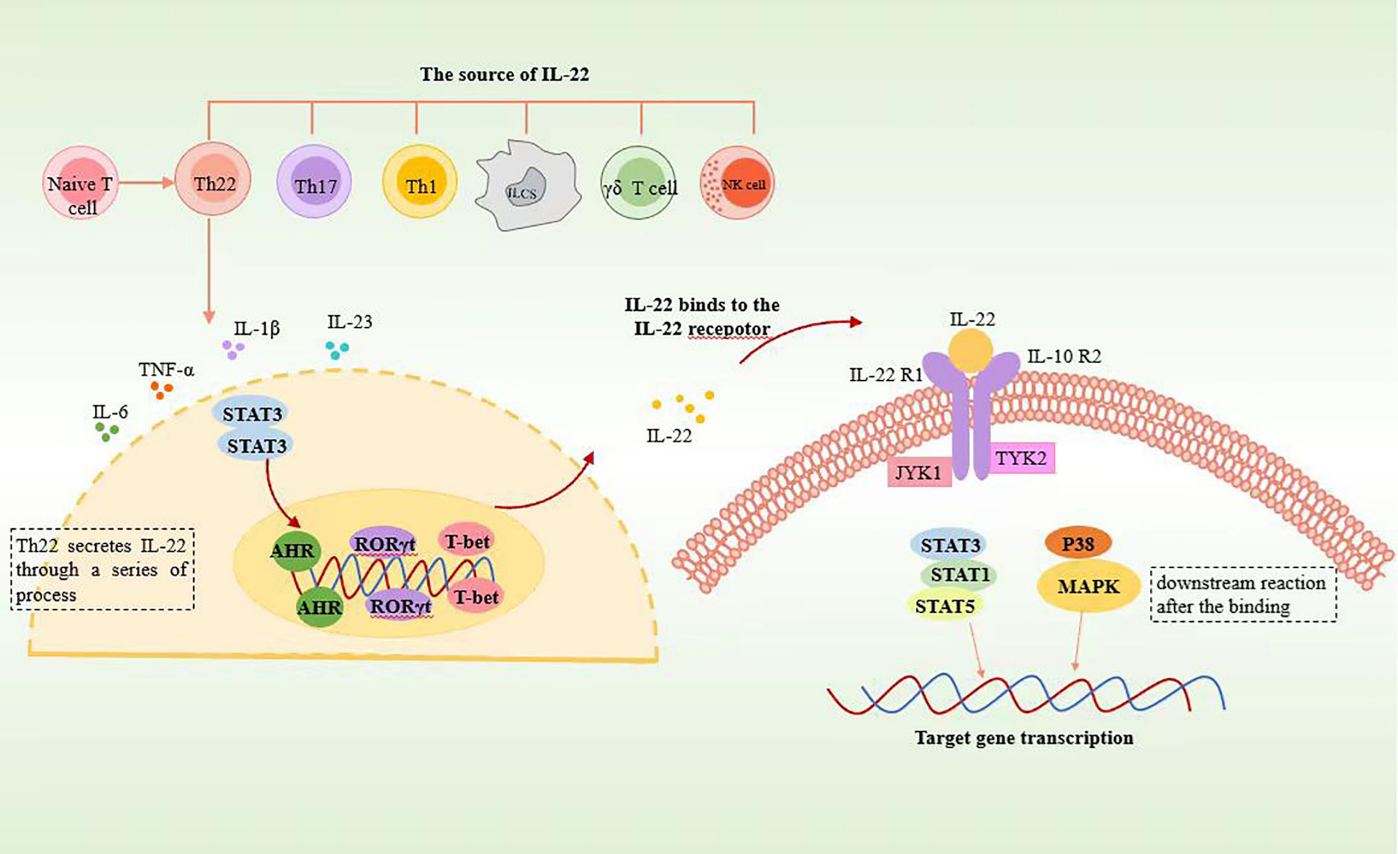
Schematic representation of Th22 cell secreting IL22. Th22 is differentiated from CD4^+^ naïve T cells. Th22 secretes IL-22 through the STAT3 pathway, in which AHR, RORγt, and T-bet play significant roles. After binding to IL-22 receptors, IL-22 induces phosphorylation of JAK1 and TYK2 through STAT3, STAT1, STAT5, and p38 MAPK pathways and then activates downstream reactions.

The involvement of the aryl hydrocarbon receptor (AHR) in inducing Th22 cell differentiation and IL-22 secretion has been well established (6, 9, 11). Additionally, RORγt has been identified as a promoter of Th22 cell differentiation, although its effect is less pronounced compared to its role in Th17 cells (6). The role of T-bet in this process remains debatable. Some studies suggest that T-bet can inhibit Th22 cell differentiation *in vitro*, and researchers, such as Maximilian and colleagues, have identified RORγt and T-bet as positive and negative regulators, respectively, of Th22 cells (7). However, it is important to note that there are opposing viewpoints, with some researchers arguing that T-bet and AHR synergistically promote IL-22 secretion *in vitro* (8) (**Figure 1**).

## Structure and physiology of IL-22

The secretion of IL-22 serves as the primary distinguishing characteristic of Th22 cells, and their functions are primarily mediated through the effects of IL-22. Mature IL-22 protein, like other members of the IL-10 family, is a secreted α-helical molecule. The biological effects of IL-22 are mediated by its binding to class 2 cytokine receptors, which are composed of heterodimeric complexes consisting of IL-10R2 and IL-22R1 (9, 12). Importantly, the IL-22R1 receptor is exclusively expressed in non-lymphoid tissues, including the skin, respiratory epithelial cells, digestive system matrix cells, liver, pancreas, synovial tissue, and mammary tissue. Consequently, unlike other cytokines, IL-22 does not directly regulate immune cells (13).

In addition to the cell surface IL-22 receptor complex, there exists a soluble single-chain IL-22 receptor referred to as IL-22 binding protein (IL-22BP) or IL-22RA2. IL-22BP has the ability to antagonizes IL-22 by occupying the binding site of IL-22 to IL-22R1 (14). Consequently, the direct binding of IL-22 to IL-22BP inhibits the actions of IL-22.

It has been confirmed that various immune cell types, including Th22, Th17, Th1, natural killer (NK) cells, γδT cells, innate immune cells, and certain non-lymphoid-like cells, are capable of producing IL-22 (15). Among these, Th22 cells are recognized as the main source of IL-22. Innate lymphoid cells (ILCs) predominantly produce IL-22 during the early stages of infection through an IL-23-dependent pathway, whereas CD4^+^ T cells secrete IL-22 during the later stages of infection via an IL-6-dependent pathway (8).

Clinical samples from patients with active UC and CD have demonstrated increased levels of IL-22 and an abundance of IL-22-expressing cells. In active UC patients, IL-22-secreting cells are primarily located in the lamina propria, while they are dispersed throughout the submucosal layer in CD patients. Despite its pro-inflammatory properties, IL-22 plays a crucial role in protecting the host from bacterial infections at barrier sites (16).

## Signaling pathway of IL-22

The JAK1/TYK2-STAT pathway serves as the primary signaling pathway for IL-22, with STAT3 playing a crucial role in this process. Minor involvement of STAT1 and STAT5 pathways has also been observed (17). The following is a detailed description of the signal transduction process.

When IL-22 binds to the IL-22R1-IL-22R2 complex, JAK kinases become phosphorylated. This phosphorylation event triggers the phosphorylation of specific amino acid residues within the cytoplasmic region of IL-22R1, enabling these residues to bind to STAT3. Subsequently, upon recognition by the corresponding sites on IL-22R1, STAT3 undergoes phosphorylation by JAK1. Phosphorylated STAT3 then translocates into the cell nucleus, where it regulates gene expression. The ultimate impact of IL-22 signaling is the inhibition of target cell differentiation or the enhancement of their proliferation. Inhibition of the IL-22-related STAT3 pathway leads to significant reductions in genes associated with apoptosis regulation (mcl1 and survivin), proliferation (myc, Reg3b, and Pla2g5), and wound healing (Smo). This suggests that the STAT3 signaling pathway in epithelial cells plays a role in the regulation of mucosal wound healing (17) (**Figure 1**).

IL-22 and IL-10 exhibit both similarities and differences in their signaling pathways. While both cytokines can activate STAT3 and induce its tyrosine phosphorylation, there are notable distinctions between them. IL-22, unlike IL-10, can trigger the activation of the ERK, JNK, and p38 MAPK pathways. This indicates that IL-22 has the ability to stimulate additional signaling cascades beyond STAT3 activation. Furthermore, IL-22 can also induce the serine phosphorylation of STAT3 through the MAPK-independent pathway. This serine phosphorylation of STAT3 represents another unique feature of IL-22 signaling, further distinguishing it from IL-10 (18).

## Intestinal mucosal barrier

The intestinal mucosal barrier comprises three main layers: the mucus layer, the epithelial cell layer, and the immune cell layer (19). The mucus layer, serving as the first line of defense for the intestinal barrier, is primarily composed of highly glycosylated mucins. It forms a gel-like mesh structure on the intestinal epithelium, preventing direct contact between bacteria and the epithelium while also influencing the gut microbial community. The transport of molecules across intestinal epithelial cells (IECs) is regulated by various junctional complexes, with the most important ones being tight junctions (TJs), adherens junctions (AJs), and desmosomes. TJs, located at the apical region, are adhesive complexes responsible for sealing intercellular gaps. They consist of transmembrane proteins (such as claudins and occludins), peripheral membrane proteins (such as occludens, ZO-1, and ZO-2), and regulatory proteins. AJs are positioned below TJs and play a crucial role in the assembly of TJs. AJs, along with desmosomes, provide robust adhesive connections that contribute to maintaining epithelial integrity (20).

The intricate functionality of Th22 cells and IL-22 has led to conflicting claims regarding their effects on mucosal barriers (**Table 1**).

**Table 1:** Some points and related references on the role of IL-22 in the intestinal mucosal barrier.

Mucosal barrier	Opinion	Reference
chemical barrier	IL-22 reduces the expression of MUC2	Patnaude et al., 2021 (21)
	IL-22 had no effect on MUC2 expression	Zha et al., 2019 (22)
	IL-22 increases the production of antimicrobial peptides	He et al., 2022 (23)
physical barrier	IL-22 has a positive effect by upregulating claudin-2	Ahmad et al., 2014 (24)
	IL-22 has a negative effect by upregulating claudin-2	Wang et al., 2017 (25)
biological barrier	IL-22 promotes the proliferation of epithelial cells	Patnaude et al., 2021 (21); Lindemans et al., 2015 (26)
	IL-22 had no effect on epithelial cells	He et al., 2022 (23)

## Role of Th22 and IL-22 in mucosal barrier

### IL-22 increases mucin secretion

MUC1 is a critical component of mucins, and the proper glycosylation of MUC1 is essential for maintaining the functionality of the mucus barrier. Hence, the integrity of the mucus barrier depends not only on the expression of MUC1 but also on the complete glycosylation of MUC1 (27). MUC2, on the other hand, reduces direct contact between bacteria and epithelial cells and can also regulate the abundance and diversity of the gut microbiota. In MUC2 knockout mice, which lack the MUC2 gene, higher levels of IL-22 are expressed, and the IL-22-STAT3 pathway plays a vital role in maintaining mucosal barrier homeostasis in these mice. This suggests a compensatory regulatory response (28).

The current literature on the impact of IL-22 on mucin composition presents conflicting results. One study indicates that IL-22 does not increase the number of cells expressing MUC2 and has no direct effect on MUC2 expression (22). However, another published study suggests that IL-22 induces the mRNA expression of MUC1, MUC4, and MUC13 while reducing the mRNA expression of MUC2 (21). The discrepancies in these findings may be attributed to differences in experimental methodologies. It is worth noting that MUC2 polysaccharides can be degraded by intestinal bacteria, and there are other factors influencing MUC2 expression. Consequently, there may be variations between *in vivo* and *in vitro* experiments (29). Due to these contradictory statements, it is challenging to draw a definitive conclusion regarding whether the mucosal changes mediated by IL-22 are beneficial for patients with IBD.

### IL-22 increases the production of antimicrobial peptides

Antimicrobial peptides, which include defensins, S100 proteins, and REG family proteins, are secreted by Paneth cells and are abundant in the intestinal mucus (30). They bind to mucus and mucins, acting as a defense mechanism against pathogen invasion. IL-22 plays a role in inducing Paneth cell differentiation through the PI3K/AKT/mTOR axis (23). By stimulating stem cells, IL-22 promotes the expression of defense genes, such as REG1A, REG1B, and DMBT1, leading to increased levels of RegIIIβ and RegIIIγ. Consequently, this results in the production of antimicrobial peptides by IECs (30, 31). Furthermore, IL-22 has been shown to influence the composition and structure of the gut microbiota (32). While the clinical application of antimicrobial peptides as therapeutic agents is not yet a reality, these studies provide potential opportunities for the development of antimicrobial peptides as novel antibiotics and bacterial modulators for the treatment of IBD.

### IL-22 affects TJs

Changes in the composition of TJ proteins can have two distinct effects on barrier function. Firstly, it can lead to increased diffusion of water and solutes into the intestinal cavity, resulting in diarrhea and the elimination of most intestinal pathogens. Secondly, it can enhance the permeability of intestinal pathogens, thereby triggering and sustaining immune responses (33). Claudins, which are the primary transmembrane proteins of TJs, primarily regulate the permeability of the barrier structure. Alterations in their expression can impact signaling pathways. While claudin-2 is upregulated by IL-22, the expression of other TJ proteins like claudin-1, ZO-1, and ZO-2 remains largely unaffected (21). The upregulation of claudin-2 elevates epithelial permeability (25), which is believed to promote inflammation. However, recent research has revealed the protective role of claudin-2, indicating that its upregulation serves as a positive defense mechanism. Studies have demonstrated that claudin-2 not only inhibits colitis-induced cell death but also suppresses colitis-induced immune activation and signal transduction (24). While it is generally accepted that the upregulation of claudin-2 and the subsequent increase in intestinal permeability contribute to inflammatory responses, its role extends beyond this. Claudin-2 also regulates epithelial cell proliferation (34), indicating that it may have both pro-inflammatory and anti-inflammatory functions, playing diverse roles in different contexts. The complex role of claudin-2 in maintaining intestinal mucosal homeostasis offers potential opportunities for the treatment of colitis.

### IL-22 affects epithelial proliferation through STAT3

Previous studies have demonstrated that IL-22 activates the proliferation of intestinal stem and epithelial cells via the STAT3 pathway, thereby contributing to the maintenance and repair of the mucosal barrier (21, 26, 35). However, a recent study has challenged this conclusion by providing evidence that IL-22 does not induce the proliferation and expansion of intestinal stem cells (ISCs) (23). Early investigations suggested an increase in the number of ISCs and epithelial cells with organelle-like properties, implying that IL-22 promotes IEC proliferation. However, it is important to note that the previous research relies solely on the observed increase in the proportion of ISCs and the volume of organoid epithelial cell populations. Yet, an increase in the proportion and volume of ISCs does not necessarily translate to an increase in epithelial cell proliferation. The enlargement of the volume of intestinal-like cells may be linked to the upregulation of claudin-2 expression. Consequently, further exploration is needed to determine whether IL-22 indeed promotes the proliferation and expansion of IECs and subsequently aids in the healing of the intestinal mucosa.

### Clinically relevant therapeutic approaches

Given the role of Th22 cells and IL-22 in maintaining the mucosal barrier, it can be inferred that pathways enhancing Th22 or IL-22 levels may hold therapeutic potential, particularly for IBD. Experimental approaches such as microinjection of IL-22 and the development of DNA vaccines containing IL-22 loci have demonstrated efficacy in reducing inflammatory cell infiltration into intestinal tissues in disease models (33). Furthermore, targeted therapies aimed at increasing IL-22 secretion from Th22 cells have been employed for IBD patients. For example, infliximab (IFX), a prominent anti-TNF monoclonal antibody (MAb), binds to soluble TNF and transmembrane TNF (tmTNF) with high affinity, offering an effective treatment strategy for patients with active CD. Anti-TNF therapy promotes IL-22 secretion in an AHR-dependent manner and facilitates the differentiation of Th22 cells, resulting in elevated IL-22 levels produced by CD4^+^ T cells. This evidence demonstrates that anti-TNF treatment promotes Th22 cell differentiation in CD4^+^ T cells of CD patients. Notably, TNF-alpha converting enzyme (TACE) has been found to inhibit anti-TNF-induced IL-22 production in CD4^+^ T cells, suggesting a potential link between varying TACE levels and the lack of response to IFX observed in some CD patients. If confirmed, TACE can serve as a promising target for the treatment of IBD patients who do not respond to anti-TNF therapy.

### Conclusion

Th22 cells are primarily responsible for secreting IL-22, making IL-22 the main product of Th22 cells. We propose that the *in vivo* mechanism of Th22 cells primarily operates through the actions of IL-22. IL-22 plays a critical role in preserving mucosal immunity against specific pathogens. It achieves these effects by facilitating the recruitment of neutrophils to combat bacterial invaders, promoting the repair of the mucosal barrier by stimulating epithelial proliferation and increasing the production of TJs, as well as inducing the synthesis of antimicrobial proteins, such as beta-defensins. IL-22 has a complex and significant role in the body. While it is believed to protect against colitis, it can also contribute to intestinal inflammation. However, the impact of Th22 cells on the mucosal barrier beyond IL-22 remains unclear, and further investigation is required to understand the precise role they play in the pathogenesis of UC and CD.

## Author contributions

JC: Writing-original draft. JY: Writing - Review and editing. All authors contributed to the article and approved the submitted version.
